# Uneven integration of health in China’s territorial spatial planning 2035

**DOI:** 10.1186/s12889-026-27930-z

**Published:** 2026-06-19

**Authors:** Yuhan Zhang, Jianquan Cheng, Lian Duan

**Affiliations:** 1 Information Center, Ministry of Natural Resources, Beijing, China; 2 Technology Innovation Center of Territorial & Spatial Big Data, Ministry of Natural Resources, Beijing, China; 3 Department of Natural Sciences, Manchester Metropolitan University, Manchester, UK; 4 Joint Centre for Urban Health and Security Intelligent Data Analytics, Nanning Normal University, Nanning, China; 5 School of Natural Resources and Geomatics, Nanning Normal University, Nanning, China; 6 Guangxi Natural Resources Intelligent Monitoring and Big Data Analysis Engineering Experimental Teaching Center, Nanning, China

**Keywords:** Healthy city, Territorial spatial planning, Thematic dimension analysis, Keyword network analysis, Intercity disparity

## Abstract

**Background:**

Spatial planning plays a pivotal role in shaping the health and well-being of urban populations. Since 2016, most Chinese cities have incorporated health-related elements into their Territorial Spatial Planning (TSP) 2035 Schemes as part of national healthy city initiatives. It is essential to examine how health is represented in these statutory planning documents and whether the level of attention varies across cities.

**Methods:**

We developed an analytical framework for healthy city planning by synthesizing international scholarly literature and governmental policy documents. Using this framework, the TSP 2035 Schemes of 36 major Chinese cities were examined through a mixed-method design combining thematic dimension analysis, keyword network analysis, and disparity measures.

**Results:**

All 36 cities have placed healthy city development on their planning schemes, focusing primarily on eight dimensions: (1) green and open space, (2) climate change and disaster response, (3) physical education (PE) facilities and public services, (4) environmental pollution prevention and control, (5) medical and health services, (6) elderly dimension, (7) health equity, and (8) healthy travel. Of these dimensions, green and open space along with climate change and disaster response receive the most emphasis. Notable healthy city keywords include ‘park’, ‘green space’, ‘healthcare’, ‘livability’, ‘sports’, ‘resilience’, ‘pollution control’, ‘disaster prevention’, ‘walking’, and ‘jogging’. Cross-city comparisons reveal substantial disparities: cities with higher administrative status generally articulated more extensive and more diverse health-related planning content, whereas many lower-tier cities pay relatively little attention to equity-sensitive dimensions.

**Conclusions:**

Health has become a visible component of statutory planning discourse in China, but its integration remains selective and uneven across cities. The results suggest that China’s healthy city development has reached an intermediate stage of “health promotion and education”, and in terms of health-spatial planning integration, China has moved beyond a narrow environmental health orientation, yet important gaps remain in equity-sensitive and people-centered dimensions. Recommendations include strengthening legislation to support healthy city planning, improving the openness and transparency of TSP 2035 Schemes, and more differentiated policy support across cities.

## Background

Spatial planning is crucial for promoting public health and well-being. Healthy cities prioritize the physical, mental, and social health of their residents, and they rely on effective spatial planning to shape land-use patterns, travel behavior, lifestyles, environmental exposures, and ultimately health outcomes [1–3]. Planning decisions also generate co-benefits by preventing communicable and non-communicable diseases and advancing environmental and social sustainability [4–6]. Historically, concern with the nexus between public health and urban spatial planning arose in late 19th-century Europe, aimed at addressing the poor living conditions in rapidly industrializing cities [7].

Over the past four decades, scholarship and policy have moved beyond a narrow understanding of planning-for-health centered primarily on sanitation and hazard control [8–16]. The World Health Organization (WHO)’s Healthy Cities movement, launched in 1986, helped reframe urban health as a multidimensional concern involving physical, mental, and social well-being [8]. Subsequent studies have further demonstrated that health is shaped not only by healthcare provision, but also by urban form, active mobility, green and open space, housing, resilient infrastructure, and wider social and environmental determinants [9–16]. In this broader perspective, planning is not merely a spatial condition for health; it is an active governance mechanism that helps organize risks and opportunities across urban space.

Building on this broader understanding of health, scholarship has developed normative and conceptual frameworks for healthy urbanism, such as Healthy Cities, Health in All Policies, and THRIVES [17–19]. Collectively, these frameworks argue that urban health should be approached not as a narrow medical or environmental issue, but as a multidimensional concern shaped by governance, equity, sustainability, and cross-sectoral coordination. They have helped broaden the field by clarifying the values and principles that should guide healthy urban development. However, while these contributions offer important conceptual direction, they are less explicit about how such principles are translated into the formal rules, categories, and priorities of statutory planning systems.

This limitation becomes especially salient when health equity is placed at the center of analysis. Research on the social determinants of health has long established that health inequalities are shaped by the unequal distribution of material resources, environmental burdens, service accessibility, and political voice across social groups and places [20, 21]. In urban contexts, these inequalities are spatially manifested through differential access to parks, healthcare, public transport, clean air, safe streets, and community facilities [22–24]. Planning therefore matters not only because it can create health-supportive environments, but also because it distributes benefits and burdens unevenly. From this perspective, health-oriented planning should not be assessed solely by the presence of desirable urban attributes; it must also be evaluated in terms of distribution, accessibility, and whose needs are recognized in policy and design. Concepts such as spatial justice and proportionate universalism have reinforced this point by arguing that planning decisions are inherently distributive and political rather than merely technical [25, 26].

International scholarship has increasingly begun to examine how these ideas are incorporated into planning and policy practice. Health in All Policies has encouraged governments to recognize that decisions in transport, housing, land use, and environmental governance can shape population health far beyond the health sector itself [27]. In the United Kingdom, for example, health has been more explicitly integrated into planning guidance through frameworks linking healthy place-making with active travel, green infrastructure, livability, and the reduction of inequalities [28]. At the same time, the literature points to persistent limitations. Empirical evidence remains strongest on associations between built-environment characteristics and health outcomes, whereas research on how statutory planning systems institutionalize health agendas is still comparatively limited. Many influential frameworks are normatively ambitious, but less clear about how competing health priorities are filtered through planning routines, regulatory formats, and uneven local capacities. These gaps suggest that an important unanswered question is not only whether health is recognized in planning, but which dimensions of health become governable through planning instruments, and how unevenly this occurs across places.

This question is especially important in rapidly urbanizing contexts such as China. China’s urbanization rate rose from 26.41% in 1990 to 66.16% in 2023, reflecting a prolonged period of rapid urban expansion. Prior to the more recent policy focus on “high-quality development,” urbanization was largely quantity-driven, leading to “urban diseases” such as environmental pollution, traffic congestion, and heat island effects [29, 30]. These issues spurred interest in the Healthy Cities movement, initially geared towards reducing urban pollution and enhancing public health infrastructure [31]. After the World Health Organization launched the Healthy Cities Network, major Chinese cities such as Beijing and Shanghai joined, reinforcing China’s commitment to health-oriented planning [32]. In the early 2000s, healthcare reform and sustainable urban strategies became prominent policy goals [33], and in 2007, the Ministry of Health designated ten cities for a pilot “Healthy Cities” project [34]. A watershed moment arrived in 2016 with the “Healthy China 2030” plan, which outlined nationwide health objectives [35]. Around the same time, the Tsinghua–Lancet Commission highlighted health challenges stemming from rapid urbanization and population ageing, reinforcing the need to connect health more systematically with urban governance and planning [36].

In parallel with this health-policy shift, China’s planning system has undergone a major institutional restructuring. Since 2018, the Territorial Spatial Planning (TSP) reform has consolidated previously fragmented planning regimes, including urban planning, land-use planning, and major functional area planning, into a unified framework under the Ministry of Natural Resources, with the aim of coordinating ecological protection, urban development, agricultural land, and public service provision across multiple scales [37]. In principle, this reform creates a significant opportunity to embed health more systematically into statutory planning. City-level TSP 2035 Schemes are especially important because they translate national and provincial priorities into local spatial strategy and define the policy language through which future urban development is guided.

However, existing scholarship has not yet sufficiently examined how health is incorporated into these statutory planning schemes. Research on healthy cities in China has often focused on policy evolution, pilot programs, urban environmental management, or specific sectors such as green space or healthcare provision [38–40]. Meanwhile, broader planning studies have discussed TSP reform primarily in terms of governance integration, ecological civilization, and land regulation [41, 42]. These literatures provide important foundations, but comparatively little attention has been paid to how health-related objectives are represented in the statutory texts of city-level TSP 2035 Schemes, which dimensions of health are prioritized, and whether such incorporation is uneven across cities.

This gap matters for both theory and practice. Statutory planning documents are not merely descriptive statements of intent; they help define what is formally recognized, measurable, reviewable, and therefore more likely to be governed through subsequent implementation processes. Examining health-related content in TSP documents can therefore reveal not only whether health has entered planning discourse, but also what kinds of health concerns are considered compatible with the logic of TSP. It also allows attention to be directed to an upstream dimension of inequality: differences in how cities codify health priorities in the planning process itself, before those differences appear in the built environment or in health outcomes.

To address this gap, this study analyzes the TSP 2035 Schemes of 36 major Chinese cities. Rather than treating healthy city discourse as a uniform policy trend, we examine how health is institutionalized in statutory planning texts and whether this institutionalization is uneven. Specifically, we ask three questions: (1) Which health-related dimensions are incorporated into China’s TSP 2035 Schemes? (2) How do cities differ in the emphasis they place on these dimensions? (3) What do these differences reveal about inequality in health-oriented spatial planning?

By answering these questions, the paper contributes to ongoing debates in public health and planning in three ways. First, it shifts the analysis of healthy city development in China from general policy description to a comparative examination of statutory planning schemes as governance instruments. Second, it develops an operational framework that translates broad healthy city debates into categories suitable for systematic document analysis. Third, by combining thematic dimension analysis, keyword network analysis, and disparity measures, it demonstrates that health integration in China’s TSP system is uneven and that inequalities may be embedded not only in urban outcomes, but also in planning priorities themselves.

## Data and methods

### Conceptual framework for healthy city planning

To examine how health is institutionalized in city-level TSP 2035 Schemes, this study develops a planning-oriented conceptual framework that identifies the main pathways through which spatial planning can shape health. Building on Wang et al. [43] and others [44, 45], we propose four pathways through which spatial planning shapes health:

1. *Reducing health risks and exposures*: e.g. improving air and water quality, safe disposal of sewage and waste;
2. *Providing accessible health resources*: e.g. equitable distribution of healthcare facilities and emergency services;
3. *Promoting physical activity and social interaction*: e.g. facilitating walkability, cycling networks, and public open spaces;
4. *Ensuring health equity*: e.g. fair allocation of resources and opportunities to all social groups.

These four pathways indicate that healthy city planning is both substantive and distributive. It is substantive because it concerns what kinds of health-supportive spaces and infrastructures are planned. It is distributive because it concerns how these elements are allocated across territories and populations. This distinction is important for the present study as city-level planning documents may emphasize certain dimensions of health while neglecting others, thereby revealing specific governance priorities rather than a neutral or comprehensive health agenda.

**Fig. 1:**
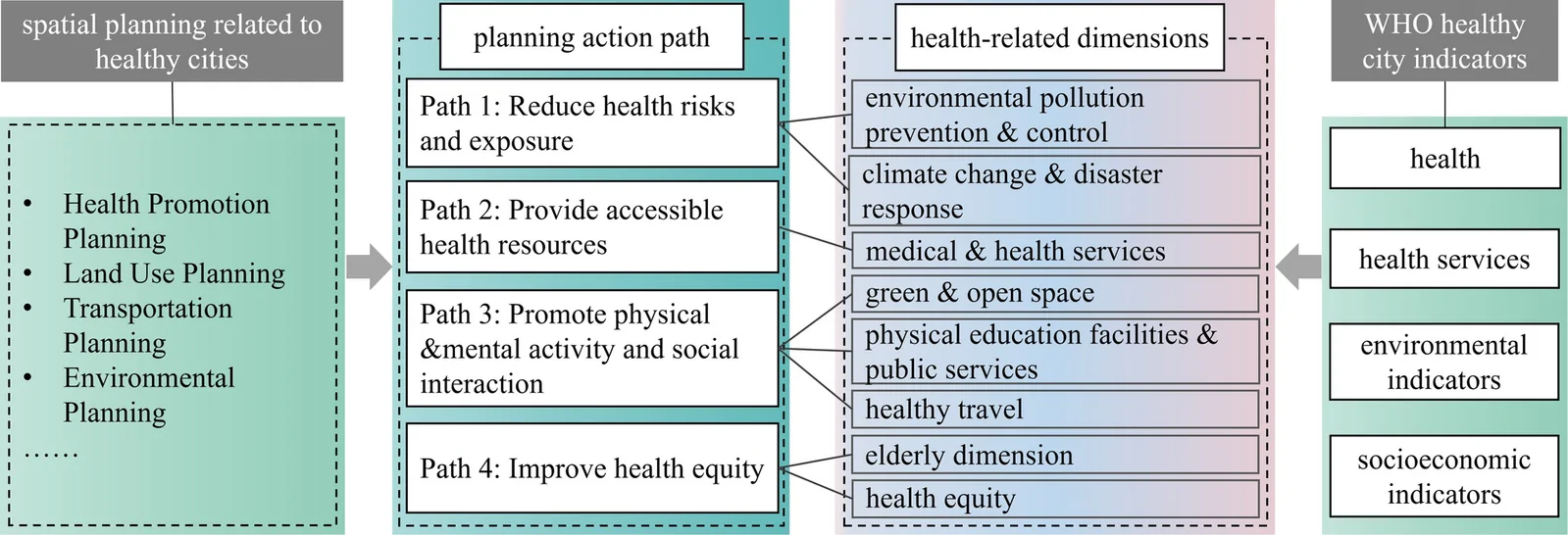
A conceptual framework. Note: Some analytical dimensions relate to more than one pathway; for visual clarity, each dimension is assigned to the pathway with which it is most directly associated

To operationalize this perspective, we synthesized international healthy city frameworks, the public health planning literature, and planning-oriented indicator systems [46–54], and identified eight health-related dimensions used in the empirical document analysis: Environmental pollution prevention & control; Climate change & disaster response; Green & open space; Medical & health services; Physical education (PE) facilities & public services; The elderly dimension; Healthy travel; and Health equity. The four pathways represent higher-level mechanisms, whereas the eight dimensions represent specific planning domains in which these mechanisms are expressed through policy language. Environmental pollution prevention & control and climate change & disaster response align primarily with the first pathway. Medical & health services correspond mainly to the second pathway. Green & open space, physical education facilities & public services, and healthy travel align most strongly with the third pathway. Elderly dimension and health equity are most closely linked to the fourth pathway (Fig. 1).

### Study sample and data sources

We selected 36 economically significant Chinese cities, including four direct-administered municipalities under the central government (Beijing, Shanghai, Tianjin, Chongqing), multiple sub-provincial cities, and additional provincial capital cities. Collectively, they represent about 26.4% of the national population and 38.6% of Gross Domestic Product [55]. These cities typically have stronger institutional capacity and policy support, making them well positioned to adopt cutting-edge healthy city measures.

**Fig. 2:**
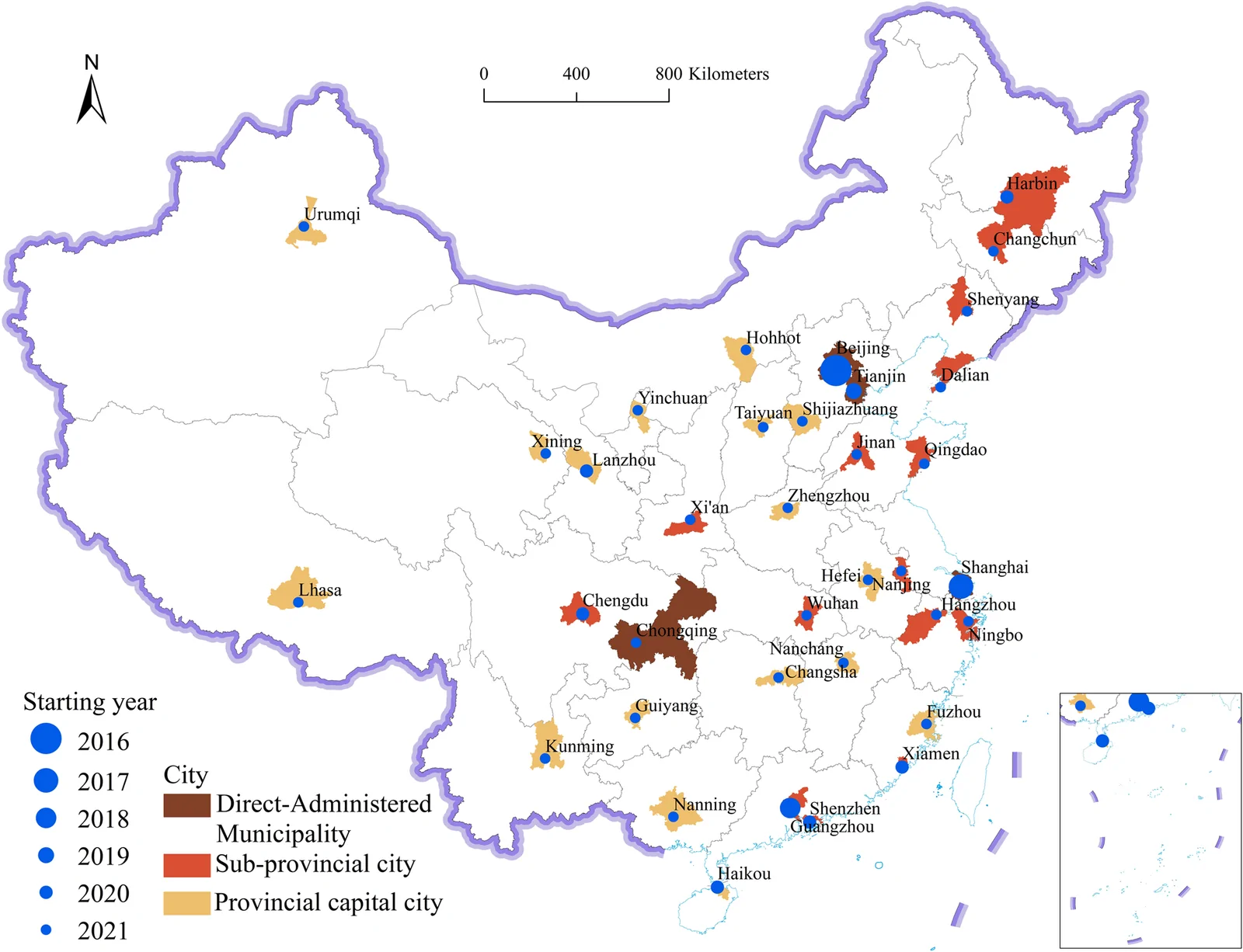
Spatio-temporal distribution of 36 cities’ TSP 2035 Schemes

All 36 cities are required to produce TSP 2035 Schemes, though their starting years vary from 2016 to 2021 (see Fig. 2). These official planning documents were downloaded from municipal Planning and Natural Resources Bureau websites. Municipalities with higher administrative status or stronger economic foundations, such as Beijing (which began drafting its TSP in 2016) and Shanghai (2017), often publish more detailed plans than cities that started later.

The analysis was conducted on the original Chinese-language texts. Where necessary, documents in PDF or image-based formats were converted into machine-readable text and then manually proofread. To improve comparability across cities, non-substantive formatting elements were removed during preprocessing, including cover pages, tables of contents, repeated headers and footers, page numbers, blank sections, and administrative notices. The resulting corpus, encoded in UTF-8 format, served as the basis for both the thematic dimension analysis and the keyword network analysis. All document preprocessing and thematic coding were conducted in Chinese in order to preserve the semantic and institutional meanings of the original planning texts. English expressions used in this study are provided for reporting purposes.

### Thematic dimension analysis

To uncover which aspects of healthy city development receive the greatest emphasis, we employed thematic dimension analysis, a qualitative research method widely used to identify, interpret, and report patterns in textual data [56, 57]. Our approach comprised three components:

1. *Preprocessing and categorization*: We filtered out non-health content, and relevant text was manually coded into nine categories—eight drawn from our framework plus a “comprehensive” category capturing broad expressions such as “sustainability”, “livability”, and “healthiness”. Coding was guided by a coding manual that defined each category, specified inclusion criteria, and established decision rules based on the substantive meaning of each text segment and its planning context. To assess coding reliability, two independent coders reviewed a 15% subsample of the text. Inter-coder reliability was assessed using Cohen's Kappa [58]. The initial Kappa value was 0.78, indicating substantial agreement. Discrepancies were resolved through in-depth discussion, a consultative process that further refined our classification criteria. The final Kappa value reached 0.85, demonstrating a high level of agreement.
2. *Aggregate review*: We first conducted an aggregate review of the full TSP corpus, identifying which health-related dimensions were most frequently highlighted.
3. *City-by-city analysis*: We then examined each city’s TSP individually to explore variations in focus and degree of detail.

The coding results were reviewed repeatedly throughout the analysis process to maintain consistency. Passages that were ambiguous or potentially relevant to more than one theme were re-examined in their full textual and planning context, and category boundaries were refined through iterative discussion.

### Keyword network analysis

To complement the thematic dimension analysis at a finer semantic level, we conducted a keyword co-occurrence network analysis of recurrent health-related terms in the TSP corpus. This procedure examines the frequency and co-occurrence of health-related keywords in order to identify prominent concepts and their associations within planning discourse.

Drawing on multiple healthy city indicator systems [49–53], we first compiled an initial list of candidate keywords, primarily in English. These terms were then translated into Chinese and contextually adapted to reflect terminology commonly used in Chinese statutory planning documents. Where multiple Chinese expressions were possible, keyword selection was guided by actual policy and planning usage and refined through iterative review of the corpus. The resulting keyword list comprised 77 terms (e.g. “park”, “green space”, “sports”, “resilience”).

Given the semantic complexity of policy documents, several steps were taken to reduce ambiguity and potential keyword misclassification. First, synonymous or near-synonymous expressions were merged to improve consistency across documents. Second, context-dependent terms were checked against their surrounding textual context to reduce irrelevant matches. Third, ambiguous expressions that could refer to multiple policy domains were manually reviewed and retained only when their use was clearly health-relevant in the planning context.

We then applied VOSviewer (version 1.6.19) [59, 60] to examine keyword co-occurrence, where two keywords were considered connected if they appeared in the same paragraph. For each keyword, we recorded: (1) frequency, defined as the number of times the keyword appeared in the corpus; (2) link strength, defined as the extent to which it co-occurred with other keywords.

After analyzing the combined corpus of all 36 TSP documents, we repeated the procedure at the individual city level to identify both overarching patterns and city-specific variation.

### Disparity analysis

To quantify intercity differences in planning attention, we calculated the coefficient of variation (CV) and Gini coefficient for each health-related dimension and for overall health-related content. These measures were used to assess how unevenly health-related planning attention was distributed across the 36 cities. We also compared average levels of health-related content across city types grouped according to administrative status.

### Analytical framework

**Fig. 3:**
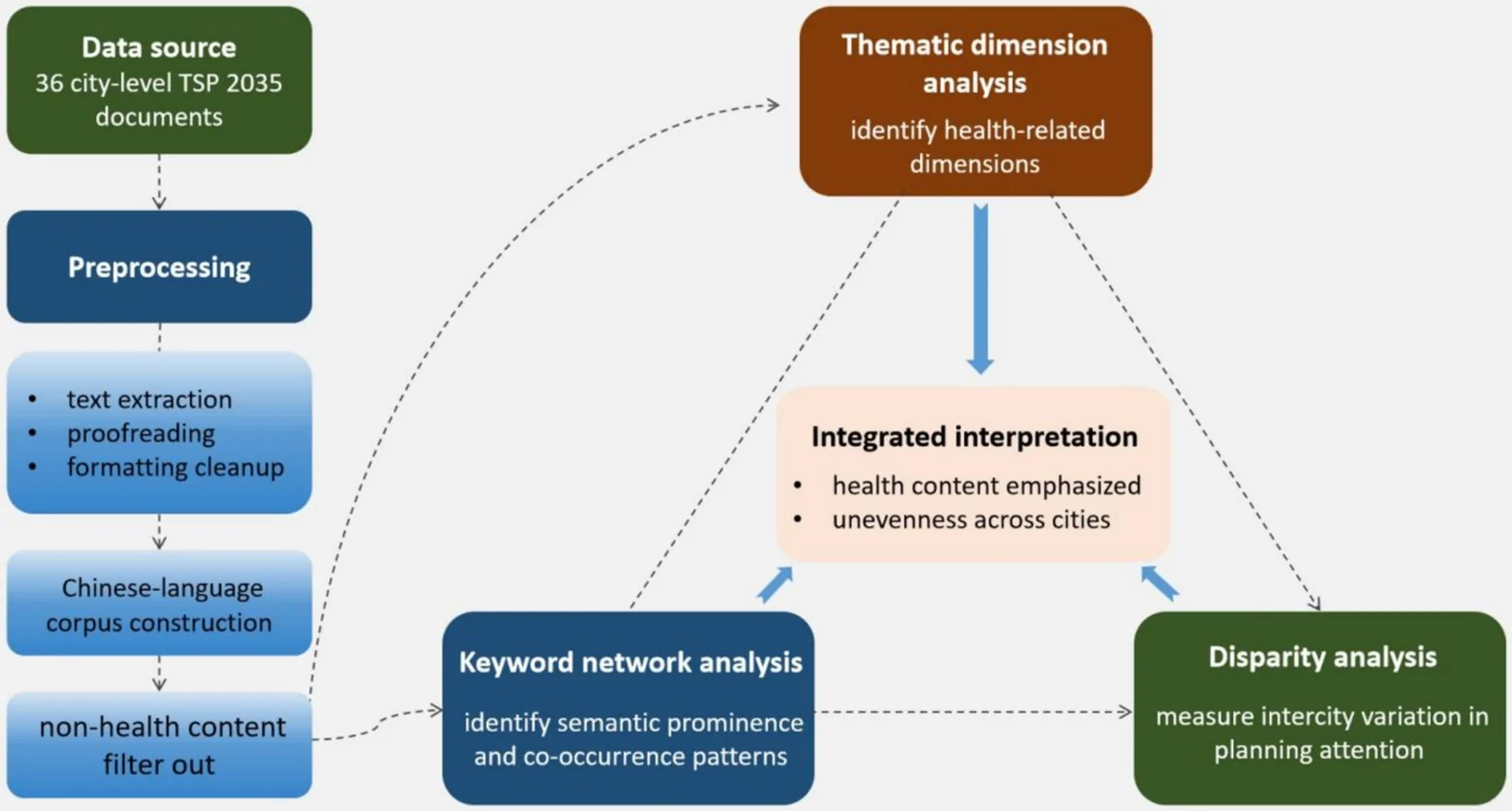
Analytical framework of the study

Figure 3 presents the study’s analytical framework. Following document collection and preprocessing, the analysis proceeded in three linked stages. First, thematic dimension analysis was used to identify and classify health-related planning content according to the eight analytical dimensions derived from the conceptual framework. Second, keyword network analysis was conducted to provide a supplementary semantic perspective on how health-related concepts were articulated and associated in planning discourse. Third, disparity analysis was used to quantify variation in planning attention across cities. Together, these methods provided a more comprehensive understanding of both the content and distribution of health-related planning discourse.

## Results

### Quantitative indicators

China’s national Urban-level Territorial Spatial Planning Guidelines specify 11 health-related indicators. However, we identified a total of 35 health-related reference items across the 36 TSP documents, including formal indicators, target values, planning targets, and target-oriented policy expressions (Table 1), implying that local governments supplemented the national baseline with additional items reflecting local priorities and planning contexts.

**Table 1:** Dimensions and disclosed health-related planning items in the TSP documents of the 36 cities

Dimensions	Indicators	Dimensions	Indicators
Environmental pollution prevention & control	Urban and rural sewage treatment rate	Elderly dimension	Beds in elderly care institutions per thousand population
	Achieving water quality standards in major rivers and lakes		Elderly care beds per thousand elder people
	Harmless treatment rate of garbage		Per capita land area for elderly facilities
	Native landfill rate		Coverage rate of community elderly facilities within a 5-min walk
	Urban and rural solid waste harmless treatment rate		Number of community elderly beds
	Environmental air quality excellent rate	PE facilities & public services	Per capita sports land area
	Proportion of clean energy consumption		Per capita sports venue area
Green & open space	Per capita park green area in built-up areas		Increase in sports venue area
	Coverage rate of park green areas and squares within a 5-min walk in the central urban area		Coverage rate of PE facilities within a 15-min walk
	Coverage rate of park green areas within a 500-m radius in built-up areas		Number of public sports facilities in each community life circle
	Urban built-up area green coverage rate		Coverage rate of community public service facilities (healthcare, elderly care, education, culture, sports, etc.) within a 15-min walk
	Area reachable by district-level greenways	Climate change & disaster response	Flood control standards in the central urban area
	Establishment or enhancement of greenways		Anti-flooding standards in the central urban area
	Length of greenways		Sponge city construction rate in built-up areas
	Index of comprehensive parks per ten thousand people		Rainwater on-site utilisation rate
			Per capita emergency shelter area
Medical & health services	Number of medical and health institution beds per thousand people	Healthy travel	Proportion of green transportation in the central urban area
	Number of infectious disease hospital beds per thousand people		Response time of emergency services in the central urban area

**Fig. 4:**
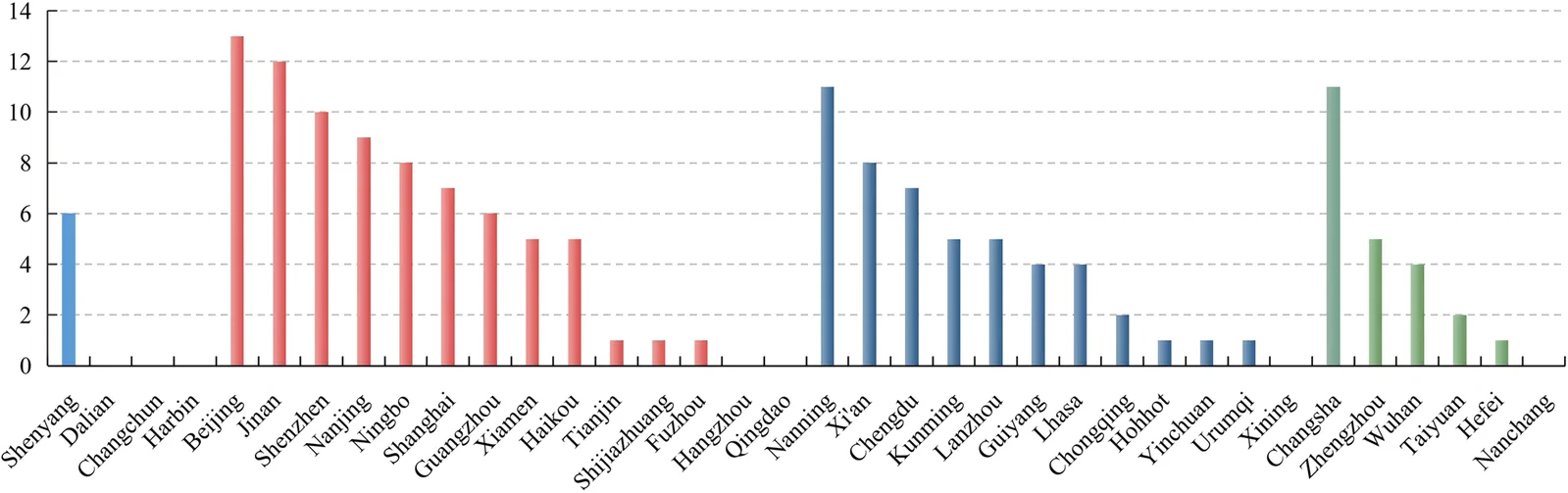
Number of indicator goals disclosed by cities

Cities varied in disclosing these indicators: while some publicized a full set of targets, others published only a few or none at all. Of the 36 cities, 7 did not disclose any health-related indicators, and 12 revealed fewer than 5. Targets also differed widely. For instance, Xi’an set a target of 20 square meters of park-green area per capita in built-up zones, whereas Nanjing set a target of 12 square meters. Eastern cities showed a tendency to publish more indicators overall (Fig. 4), likely reflecting stronger economic capacity and governance structures [61, 62].

### Thematic analysis of health-related dimensions

**Fig. 5:**
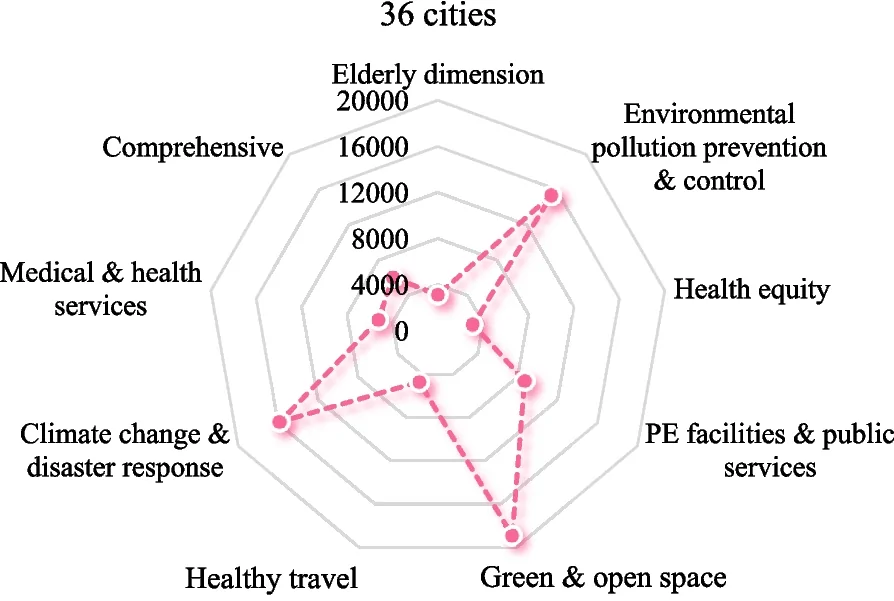
Radar chart showing word counts for each thematic dimension

The word-count-based thematic dimension analysis (Fig. 5) reveals that green & open space (4.3% of total text), climate change & disaster response (3.6%), and environmental pollution prevention & control (3.5%) dominate health-related content in the TSP documents. Meanwhile, health equity and the elderly dimension each account for just 0.7%, and collectively health-related content constitutes 18.3% of the total text across the corpus.

**Fig. 6:**
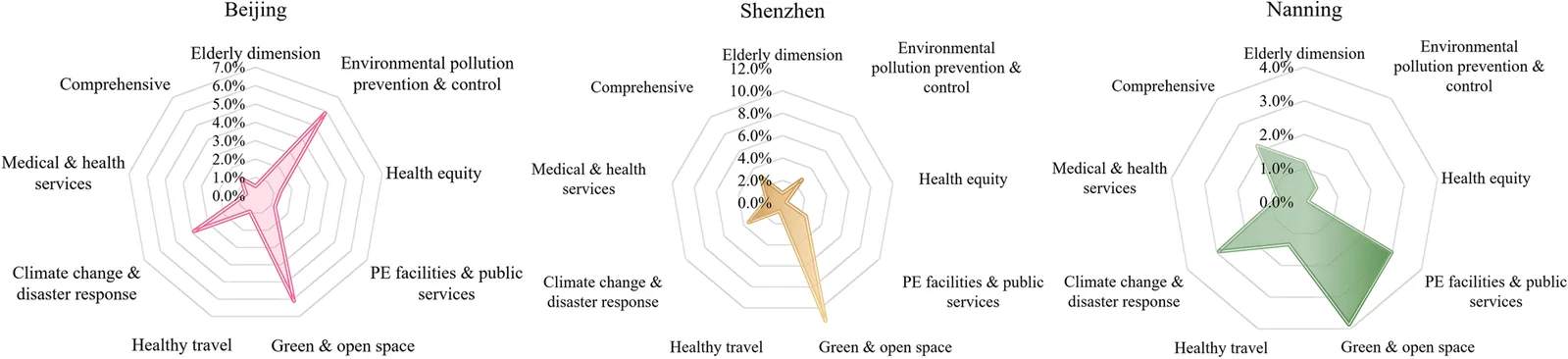
Radar chart depicting word counts for each thematic dimension in three cities (Beijing, Shenzhen, and Nanning)

When identifying which dimension each city emphasizes most, 19 cities place the greatest emphasis on green & open space, 8 on climate change & disaster response, 5 on environmental pollution prevention & control, and 4 on PE facilities & public services. For instance, Beijing focuses on three key dimensions, broadly mirroring the overall trend, whereas Shenzhen (a sub-provincial city) concentrates on green & open space, and Nanning (a provincial capital city) devotes particular attention to both green & open space and PE facilities & public services (Fig. 6).

### Intercity disparities

**Fig. 7:**
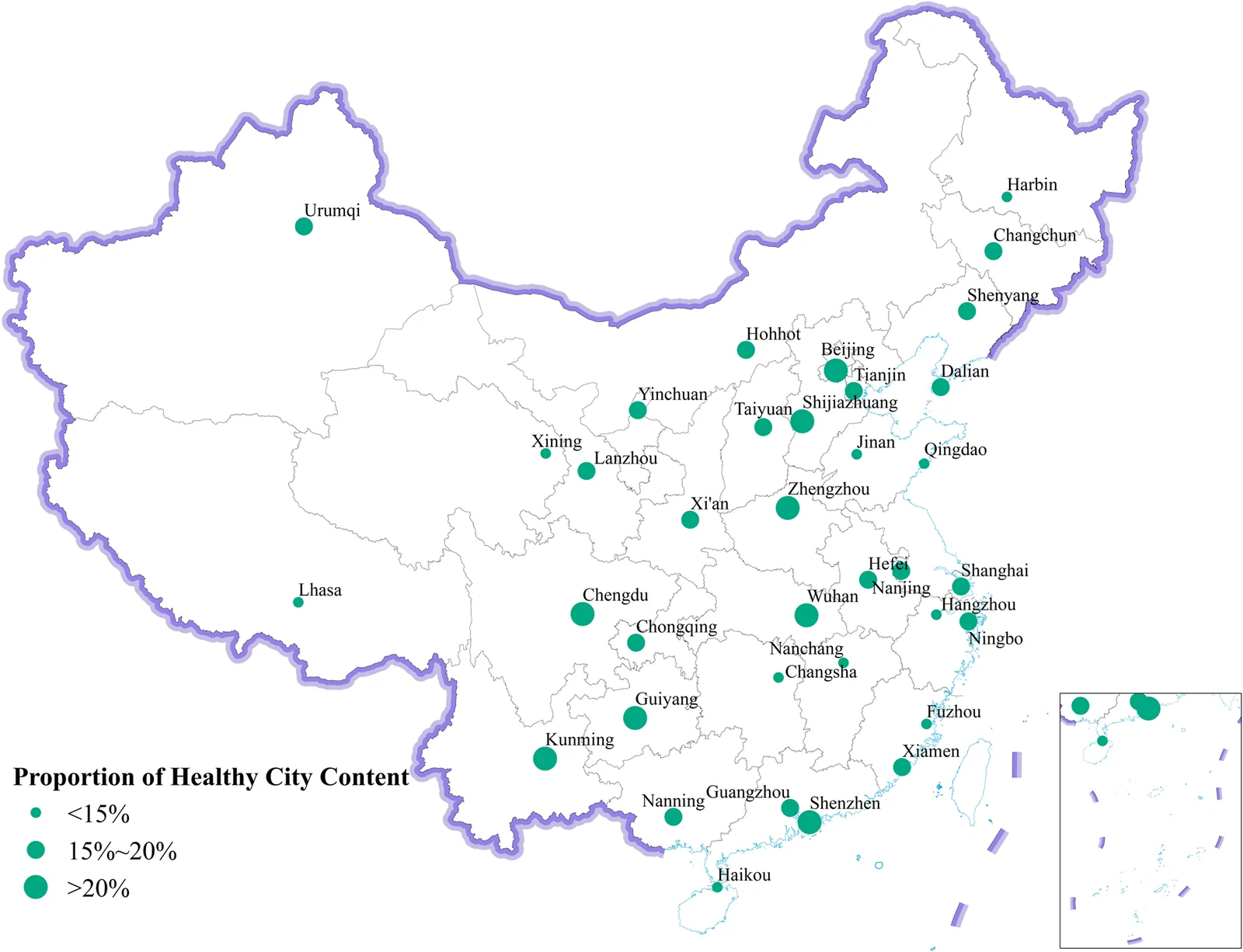
Proportion of Healthy City Content in the TSP 2035 Schemes of the 36 Cities

The share of TSP text dedicated to healthy city considerations ranges from 9% (Fuzhou) to 26% (Beijing), as illustrated in Fig. 7. Cities with higher administrative status generally devote more attention to health-related issues: direct-administered municipalities average 19.0%, sub-provincial cities 17.6%, and provincial capital cities 17.2%. Although these differences are not enormous in absolute terms, they indicate a consistent gradient across categories of city administrative status.

**Fig. 8:**
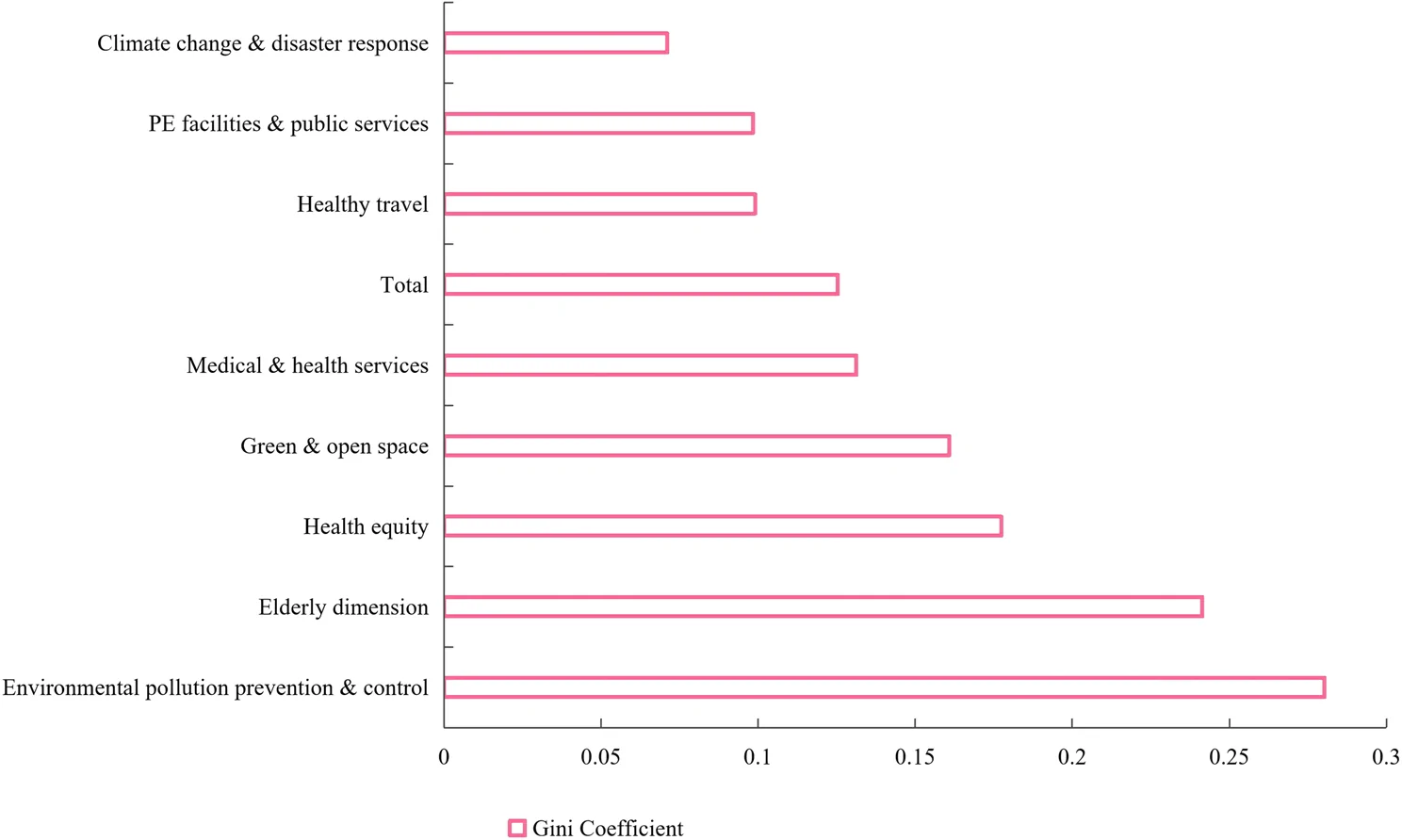
Gini coefficient for the content of each thematic dimension

Coefficient-of-variation (CV) values for each dimension also demonstrate substantial disparities: health equity shows the highest CV (127.1%), with nine cities effectively omitting this dimension. Similarly, medical & health services (76.5%) and healthy travel (74.9%) display notable unevenness. By comparison, green & open space is more uniformly emphasized (CV of 47.3%). Gini coefficients for each dimension confirm these findings (Fig. 8), with environmental pollution prevention & control and the elderly dimension exhibiting relatively higher inequality scores.

### Keyword network structure

**Fig. 9:**
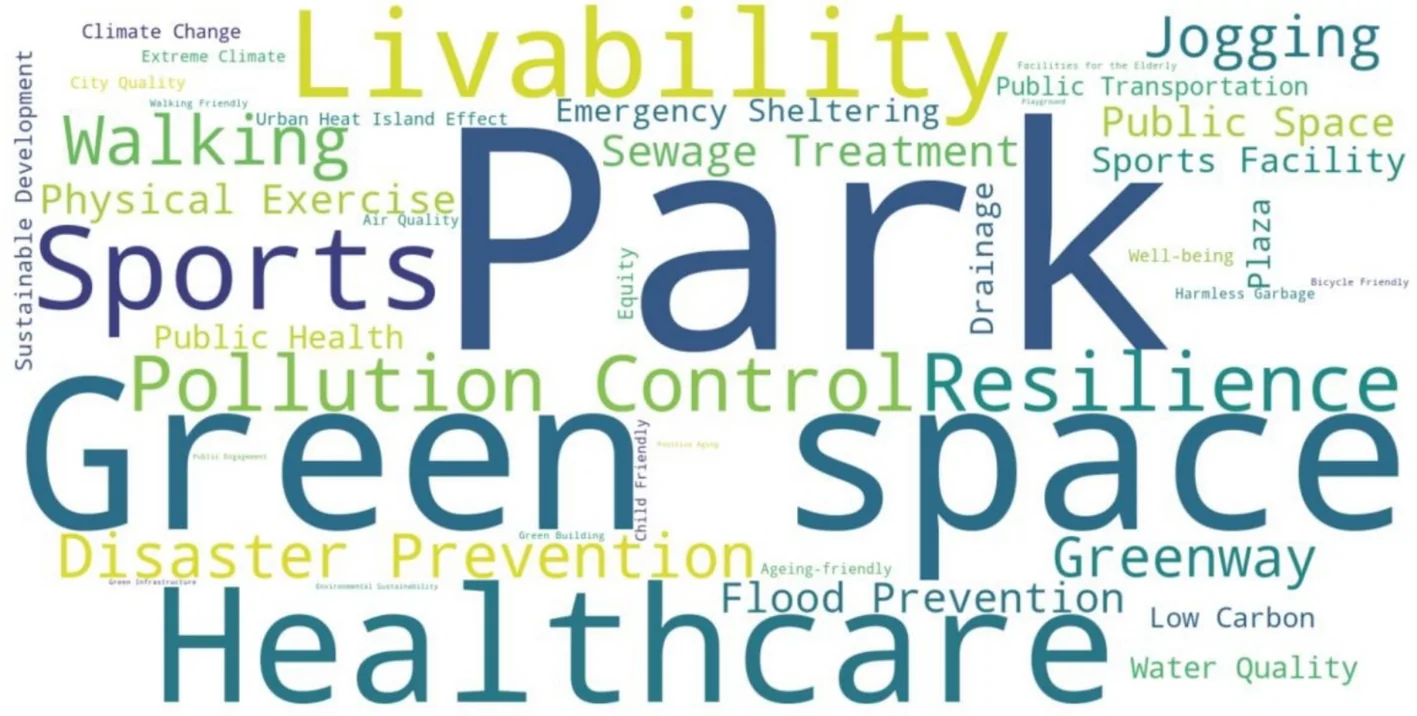
Frequency of healthy city keywords across the TSP 2035 Schemes of the 36 cities

**Fig. 10:**
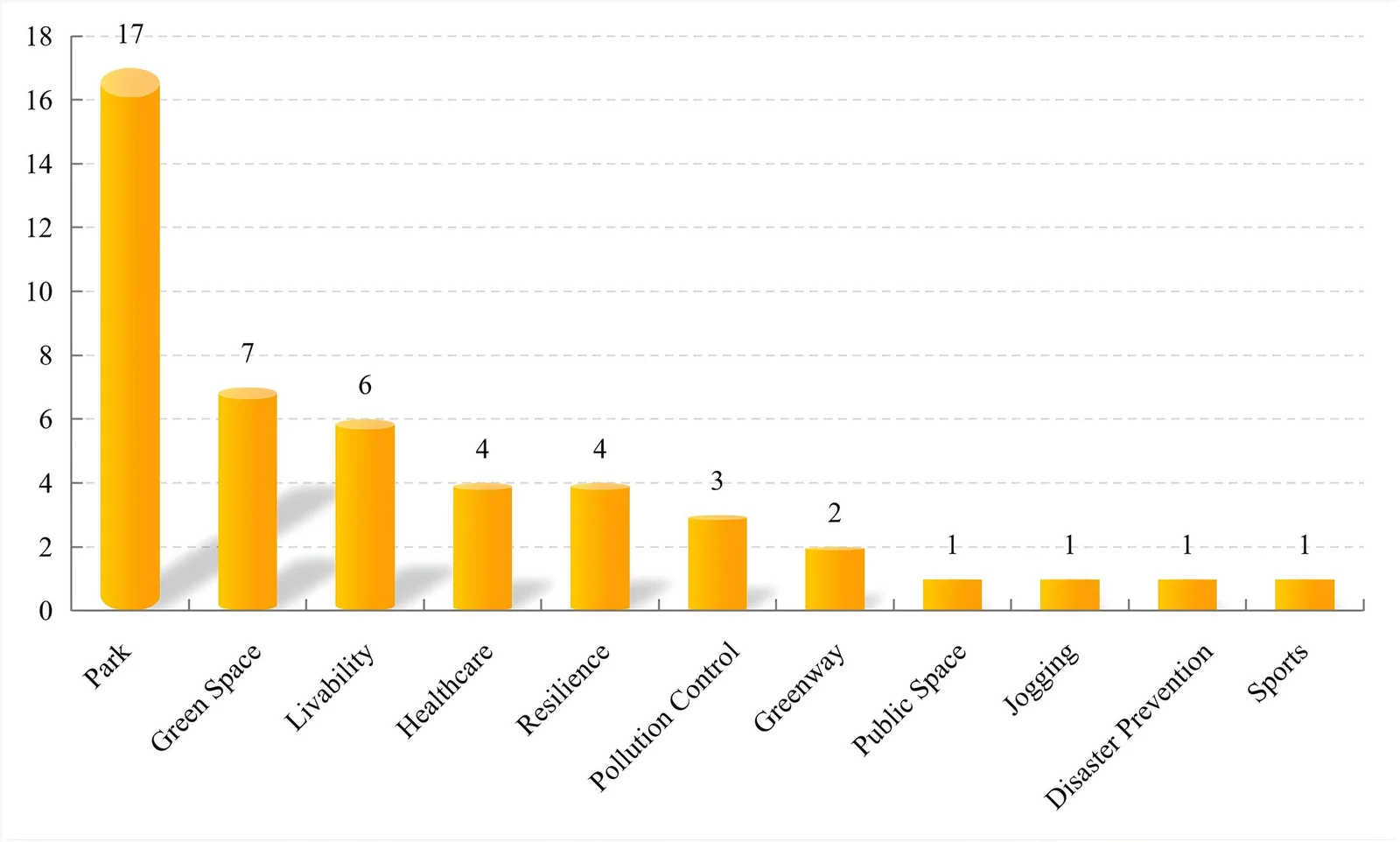
Top keywords by city and their corresponding frequencies

Across all 36 TSP documents, the top ten keywords—‘park’, ‘green space’, ‘healthcare’, ‘livability’, ‘sports’, ‘resilience’, ‘pollution control’, ‘disaster prevention’, ‘walking’, and ‘jogging’—are consistent with the thematic dimension analysis (Fig. 9) and suggest that health-related planning discourse is strongly anchored in environmental, recreational, and resilience-related concepts. ‘Park’ ranks as the most frequently used term in 17 of the cities (Fig. 10).

**Fig. 11:**
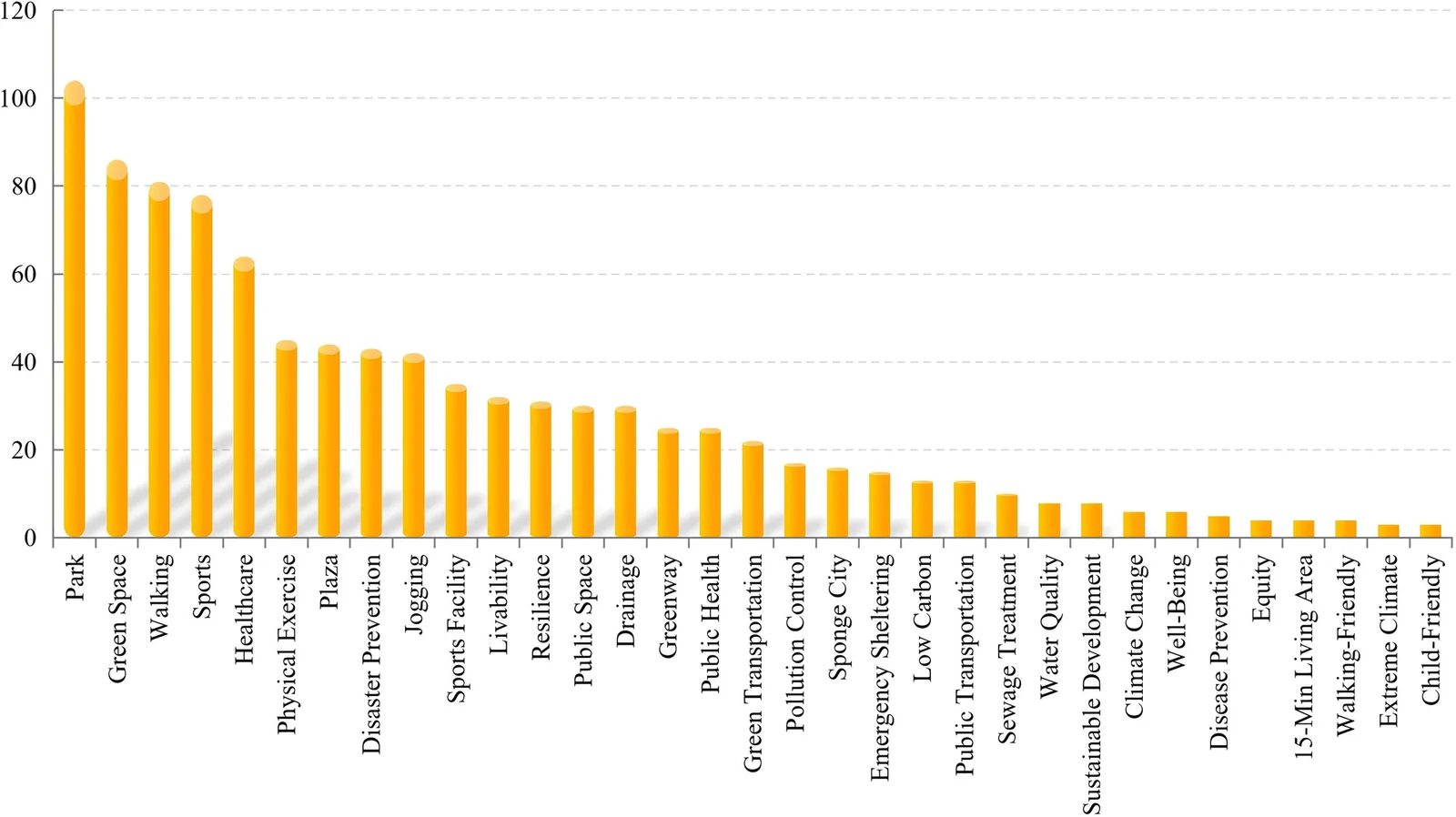
Keyword link strength across the TSP 2035 Schemes of the 36 cities

City-level keyword counts highlight Beijing and Shanghai, each with over 200 instances, followed by Nanjing and Chengdu. This suggests that higher-tier cities employ a broader array of health-related keywords. In terms of co-occurrence, high-frequency words often link to multiple other keywords (Fig. 11), suggesting stronger conceptual integration. However, certain topics (e.g. ‘facilities for the elderly’, ‘public engagement’) remain relatively disconnected, indicating that certain aspects of healthy city planning remain marginal within the broader planning discourse.

**Fig. 12:**
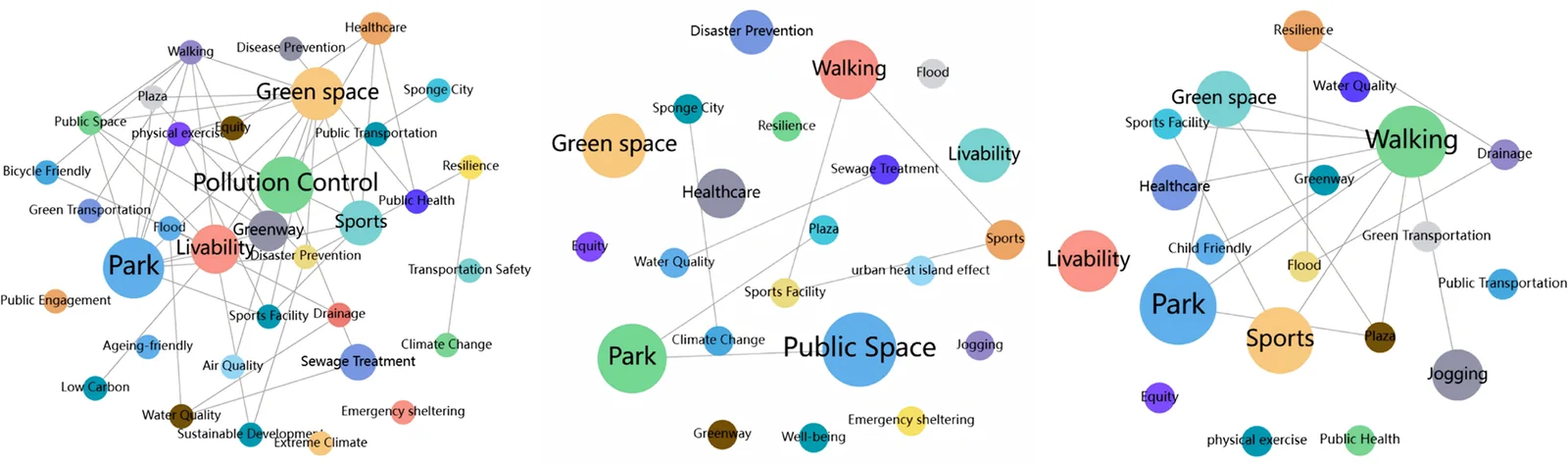
Frequency and interconnections of healthy city keywords in three cities (Beijing, Shenzhen, and Nanning)

Figure 12 visualizes the results for Beijing, Shenzhen, and Nanning. In this figure, the size of each node corresponds to the number of times a keyword appears, while the connecting lines indicate associations between keywords. The visualization reveals noticeable variations among the cities. For example, Beijing exhibits a higher frequency of healthy city keywords with more frequent interconnections. In contrast, Shenzhen, which represents sub‑provincial cities, shows relatively fewer keywords and connections, and Nanning, representing provincial capital cities, records the fewest occurrences among the three.

Analysis of the occurrence of health-related keywords across the 36 cities revealed that Beijing and Shanghai exhibit the highest frequencies, each exceeding 200 occurrences. Nanjing follows closely with 164 occurrences, and Chengdu with 73. When considering administrative levels, direct-administered municipalities stand out with an average of 118.0 keyword occurrences, significantly higher than sub‑provincial cities (45.9) and provincial capital cities (33.1). Moreover, the coefficient of variation (CV) and the Gini coefficient are notably high, at 143.6% and 0.535 respectively, indicating considerable disparity among the cities. Similarly, the overall observed link strength of 'healthy city' keywords mirrors the pattern of keyword occurrences, with direct-administered municipalities exhibiting an average link strength of 24.3, compared to 15.1 and 10.2 in sub‑provincial cities and provincial capital cities respectively. These findings underscore substantial differences in the emphasis placed on healthy city initiatives, reflecting varied public health priorities and possibly differing resource allocations across cities.

## Discussion

This study contributes to healthy city and planning scholarship by showing that the incorporation of health into China’s TSP 2035 Schemes is neither absent nor comprehensive, but selective and uneven. Health is clearly visible in the statutory planning discourse of all 36 cities, yet the form this takes is patterned: green and open space, pollution control, climate adaptation, and disaster resilience are much more consistently represented than health equity, aging-related provision, and some aspects of healthy mobility. The significance of this finding lies not simply in confirming that health has entered planning, but in revealing which dimensions of health are selectively translated into statutory planning language, and with what implications for intercity inequality. In this sense, the paper shifts the analytical focus from the binary question of whether health is included in planning to the more substantive question of how health is institutionalized, and how unevenly this occurs across places.

### From health inclusion to selective institutionalisation

A key implication of these findings is that the integration of health into planning should not be conceptualized as a binary condition of presence or absence. Much of the healthy city literature examines whether health has been incorporated into planning frameworks, often implicitly assuming that greater inclusion is inherently progressive [18, 23–26]. However, the present findings suggest that integration is better understood as a process of selective institutionalization: planning systems incorporate some dimensions of health more readily than others, while filtering, weakening, or marginalizing those that are less compatible with prevailing governance routines. This distinction matters because a planning document may adopt the language of health while still privileging only a limited subset of health concerns. International work on health-promoting spatial planning and mainstreaming health in urban planning similarly argues that embedding health requires more than rhetorical recognition; it requires sustained integration into planning processes, criteria, and governance structures [18, 63].

In the Chinese TSP documents analyzed here, the most prominent dimensions of health share several characteristics: they are spatially legible, visually demonstrable, technically measurable, and readily aligned with established planning tools such as land-use zoning, ecological control lines, infrastructure provision, and urban safety standards. In this respect, the selective prominence of these themes is not accidental; it reflects the kinds of issues that statutory spatial planning is institutionally well equipped to govern. China’s TSP reform seeks to integrate formerly fragmented planning regimes into a unified system with stronger control, coordination, and ecological governance, which makes such spatially explicit health dimensions especially compatible with the system’s core operating logic.

By contrast, health equity and aging-related provision are less visible in the plans. These dimensions are relatively harder to codify through conventional spatial allocation alone because they involve finer-grained questions of differential need, service accessibility, vulnerable populations, neighbourhood-scale distribution, and cross-sectoral coordination. They are also less amenable to showcase-style representation than park systems, resilience corridors, or pollution targets. The document evidence suggests that current TSP practice tends to privilege a territorially visible and administratively governable model of health while giving weaker and less consistent attention to more redistributive, equity-sensitive, and socially differentiated dimensions. This interpretation is consistent with broader healthy planning literature, which has increasingly emphasized that a fully health-promoting planning approach must address equity, inclusion, and active living rather than only environmental quality [23–26].

This argument has theoretical importance because it reframes healthy city planning as a question of institutional selectivity rather than normative checklist completion. In other words, the key issue is not merely whether plans mention parks, healthcare, or resilience, but whether planning institutions are capable of incorporating health as a broad, distributive, and people-centered governance principle. The Chinese case therefore contributes to wider debates in planning theory by illustrating how statutory planning systems do not passively reflect external agendas; they actively filter and translate those agendas according to their own administrative capacities, spatial tools, and scalar priorities.

### Planning inequality as an upstream dimension of urban health inequality

Most research on urban health inequality focuses on downstream outcomes: unequal exposure to pollution, unequal access to healthcare, unequal availability of green space, or unequal opportunities for physical activity [64, 65]. The present analysis suggests that inequality may also emerge upstream, in the uneven incorporation of health priorities into statutory planning documents. This is important because statutory plans are not neutral mirrors of existing preferences; they are governance instruments that help define what is legitimate, measurable, fundable, and governable within future urban development. If some cities devote little attention to equity or ageing in their formal planning schemes, these issues may also receive less attention in subsequent implementation, monitoring, and resource allocation. From this perspective, intercity variation in TSP content may indicate not only discursive difference but also an earlier layer of inequality within the policy process itself.

Seen in this light, the paper extends healthy city research beyond outcome inequality toward policy inequality or institutional inequality. It suggests that the uneven geographies of health may be partly rooted in uneven capacities to define health as a planning priority in the first place. This is one of the paper’s main conceptual contributions: inequality in healthy urban development should be studied not only as a result of uneven services and environments, but also as a consequence of uneven attention, codification, and strategic incorporation within planning systems.

### Intercity disparities, planning capacity, and state selectivity

The results show clear intercity disparities in both the volume and diversity of health-related planning content. Direct-administered municipalities and, to a lesser extent, sub-provincial cities generally disclose more health-related indicators, use denser health vocabularies, and devote more textual space to health. One plausible interpretation is that administrative status functions, at least partly, as a proxy for broader differences in planning capacity. Higher-tier cities often possess stronger fiscal resources, more specialized expert teams, greater analytical infrastructure, and more experience in translating central policy agendas into detailed statutory planning content. They may also face stronger demonstration pressure to align with national priorities and to act as exemplary sites of policy innovation. These are plausible explanatory mechanisms rather than directly tested causal claims, but they help make sense of the observed gradient.

If higher-tier cities are better able to articulate health in diverse and detailed planning terms, while lower-tier cities rely on thinner or more standardized formulations, then China’s territorial planning reform may be generating a differentiated landscape of policy capacity. This raises an important governance question: can a national strategy of Healthy China and integrated territorial planning deliver consistent health-sensitive planning across cities with uneven resources, expertise, and institutional visibility? The present findings suggest that without stronger baseline requirements and support mechanisms, the answer may be only partially.

### Interpreting China’s TSP practice in a broader conceptual context

**Fig. 13:**
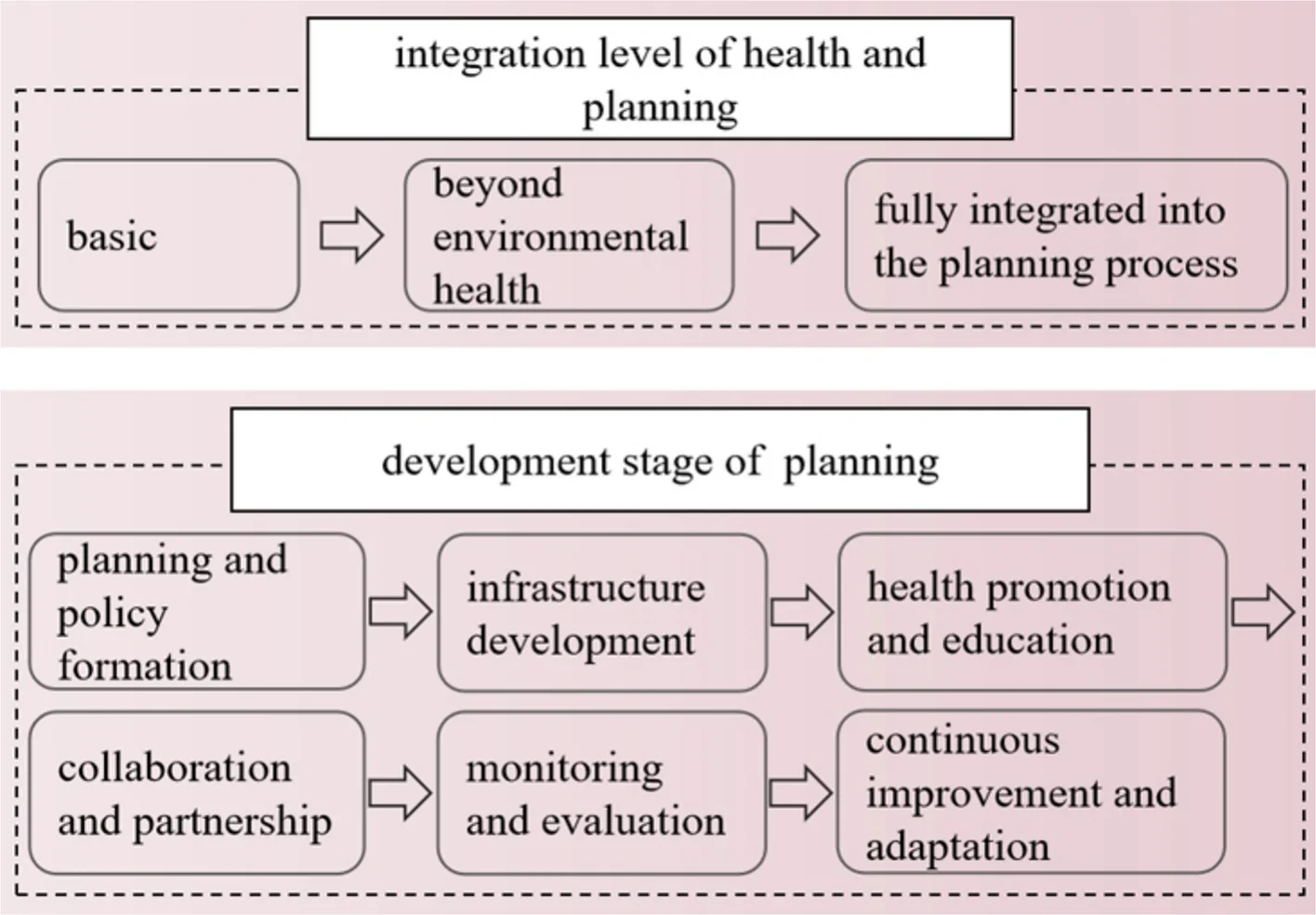
Levels of health–planning integration and developmental stages of healthy city planning

To situate these findings within a broader context, we refer to two conceptual frameworks (Fig. 13). First, levels of health–planning integration, which range from meeting basic environmental sanitation needs to fully embedding health considerations in all planning processes. Second, stages of healthy city planning, spanning from policy formation through implementation, education and outreach, intersectoral collaboration, monitoring, and continual refinement [66–70].

With regard to the degree of health–planning integration, our analysis suggests that China is currently at the “beyond environmental health” level, signifying partial but not complete integration of health within spatial planning. The prominence of environmental and resilience-oriented themes shows clear progress beyond a narrow sanitation model, yet the limited integration of equity-oriented and socially differentiated health concerns indicates that the shift toward full health-sensitive planning remains incomplete.

In terms of the development stage of healthy city planning, China is currently at the stage of “health promotion and education” compared with contexts in which health has been routinely monitored through established policy frameworks [71, 72]. Although many Chinese cities have recently begun implementing healthy city strategies—such as creating more green space and sports facilities—a comprehensive evaluation process with iterative feedback has not yet materialized. Most TSP 2035 Schemes were only released around 2021, and the mandated five-year evaluations have not yet commenced.

### Policy implications and future directions

The findings have several practical implications for the next phase of health-sensitive territorial planning in China.

1. *Strengthen legislation*: Formally incorporate healthy city considerations into the legal and regulatory framework for TSP at all levels. A minimum set of required health-related planning content, indicators, review criteria, and monitoring benchmarks for city-level schemes should be defined in planning content. Healthy China 2030 and related policy statements already emphasize health integration across sectors; the challenge is to convert that strategic commitment into more consistent statutory requirements.
2. *Improve openness*: Require that local governments disclose all relevant indicators, targets, and benchmarks, facilitating evaluation and civic participation.
3. *Balance neglected elements*: Provide incentives or directives for prioritizing underemphasized dimensions such as health equity, healthy travel, and facilities for the elderly.
4. *Offer differentiated support*: Support mechanisms for lower-capacity cities should include standardized guidance, indicator templates, shared data platforms, technical training, and intercity learning networks. Demonstration pilots in more advanced cities may also help, but diffusion should be accompanied by practical tools that lower-capacity cities can actually use in drafting and revising their schemes.

### Limitations

First, this study focuses on 36 large cities and may not capture the circumstances of smaller or less developed municipalities. Second, our data are limited to publicly available TSP documents; unpublicized or informal healthy city efforts could therefore be overlooked. Third, the study evaluates the representation and disclosure of health-related issues in statutory planning texts rather than the implementation of planning measures or their effects on health outcomes. Nonetheless, the analysis illuminates important trends and imbalances in how Chinese cities are integrating health considerations into official planning.

## Conclusions

This study examined how health-related planning content is represented in the TSP 2035 Schemes of 36 major Chinese cities. The findings show that:

1. Health has become a visible component of China’s statutory planning discourse, but its integration remains uneven and selective.
2. Green & open space, climate change & disaster response, environmental pollution prevention & control, and healthcare are top priorities. Whereas health equity, healthy travel, and the elderly dimension often receive less attention.
3. Significant disparities exist among the 36 cities, with higher-tier cities generally disclosing more indicators, use richer health-related vocabularies, and devote more textual attention to health than lower-tier cities.
4. Public disclosure of targets and indicators remains inconsistent.

These findings suggest that China’s current TSP practice has moved beyond a narrow environmental sanitation perspective, but has not yet achieved a fully balanced, equity-sensitive, and socially differentiated integration of health into planning. Future progress will depend not only on stronger legislation and greater transparency, but also on more differentiated support for cities with weaker planning capacity and on a broader redefinition of health as a core principle of territorial governance.

## Data Availability

The datasets supporting this study are publicly available from the sources listed in the Appendix. The analytical corpus was originally compiled on 30 June 2023. During manuscript revision, the source list was rechecked and updated to confirm publication details and link accessibility, and some Appendix entries therefore reflect bibliographic information updated after the original data collection date. Derived datasets generated during the current analysis are available from the corresponding author upon reasonable request.

## Appendix

The websites of 36 cities’ TSP 2035 Schemes (All URLs were last accessed and confirmed to be valid on March 12, 2025):

Beijing: https://www.beijing.gov.cn/gongkai/guihua/wngh/csztgh/201907/t20190701_100008.html

Tianjin: https://ghhzrzy.tj.gov.cn/zmhd_143/jcyjzj/202109/t20210923_5608995.html

Shijiazhuang: https://www.sjz.gov.cn/zfxxgk/columns/77594014-91df-4b39-b76a-d81d651f627b/202410/15/84e965a5-0ef7-4538-95cd-d0779d860b0c.html

Taiyuan: https://ghzy.taiyuan.gov.cn/pqgs/20221028/30000899.html

Hohhot: http://zrzyj.huhhot.gov.cn/zwgk/tzgg/202209/t20220905_1366453.html

Shenyang: https://www.shenyang.gov.cn/zmhd/yjzj/yjzj/202111/t20211102_555451.html

Dalian: https://zrzy.dl.gov.cn/art/2021/9/6/art_3083_1866820.html

Changchun: http://www.changchun.gov.cn/hd/tczj/202210/t20221025_3075792.html

Harbin: https://www.harbin.gov.cn/haerbin/c104710/202107/c01_145700.shtml

Shanghai: https://www.shanghai.gov.cn/nw42806/index.html

Nanjing: https://mp.weixin.qq.com/s/vHuC5dbinLXxXMrW8mKNIg

Hangzhou: http://ghzy.hangzhou.gov.cn/art/2021/5/31/art_1229369034_3877101.html

Ningbo: https://zgj.ningbo.gov.cn/art/2022/12/11/art_1229630397_49661.html

Hefei: https://zrzyhghj.hefei.gov.cn/public/13081/107504816.html

Fuzhou: https://zrzyt.fj.gov.cn/zwgk/xwdt/zrzyyw/202108/t20210823_5674281.htm

Xiamen: https://mp.weixin.qq.com/s/Si1agAKGukz-0RvOg8IpZg

Nanchang: http://bnr.nc.gov.cn/ncszrzyj/tzgg01/202210/b44f4e9e86ac4a0695a091b8e6fd5a20.shtml

Jinan: http://nrp.jinan.gov.cn/art/2024/9/3/art_43695_5015610.html

Qingdao: http://zrzygh.qingdao.gov.cn/zxfw/zrzyghggzyxx/ghgl/ghcajpzjg/csztgh/202204/t20220402_5165591.shtml

Zhengzhou: https://zrzyhghj.zhengzhou.gov.cn/ztgh/6752651.jhtml

Wuhan: https://www.wuhan.gov.cn/zwgk/wgk/jcgk/ygk/202107/t20210714_1738706.shtml

Changsha: http://zygh.changsha.gov.cn/zfxxgk/zwyw/xwdt/bjdt/202112/t20211229_10420658.html

Guangzhou: https://ghzyj.gz.gov.cn/zwgk/ztzl/gtkjgh/gzxx/content/post_6484475.html

Shenzhen: https://www.sz.gov.cn/szzt2010/wgkzl/jcgk/jcygk/zdzcjc/content/post_8858880.html

Nanning: https://zrzyj.nanning.gov.cn/zwgk_57/tzgg/gggs/t4965247.html

Haikou: https://mp.weixin.qq.com/s/FXwq2Zn39mGAAqQlLbv0ZA

Chongqing: https://www.cq.gov.cn/zwgk/zfxxgkml/zdjcygk/zdjcyjzj/202105/t20210527_11479457.html

Chengdu: https://mp.weixin.qq.com/s/IXCTyb78NnvB6kDSf9ctjw

Guiyang: https://zyghj.guiyang.gov.cn/newsite/ztzl/yhyshj/bsfw/ghcg/gtkjgh/202404/t20240409_84169930.html

Kunming: https://www.km.gov.cn/c/2022-11-10/4634396.shtml

Lhasa: https://zrzyj.lasa.gov.cn/zrzyj/c100746/202304/dd250d2fa79b4d00b46549e4f20019db.shtml

Xi’an: https://zygh.xa.gov.cn/xwzx/tzgg/636a34ebf8fd1c4c21276a2a.html

Lanzhou: https://mp.weixin.qq.com/s/fFhDFADM9SqNHUq37mfKMQ

Xining: https://zygh.xining.gov.cn/sy/tzgg/202404/t20240403_201785.html

Yinchuan: https://www.yinchuan.gov.cn/zmhd/yjzj_7926/zdxzjc_72837/202211/t20221117_3847509.html

Urumqi: https://www.urumqi.gov.cn//wlmqs/c119201/202212/cb52fa327b2c47978cf36679b99f2dfe.shtml

The full list of the 77 health-related keywords:

15-Min Living Area; Active Commuting; Active Living; Active Travel; Ageing-friendly; Air Quality; Bicycle Infrastructure; Bicycle-Friendly; Child-Friendly; City Quality; Climate Change; Collaborative Governance; Community Engagement; Community Garden; Digital Health; Disability Friendly Design; Disaster Prevention; Disease Prevention; Drainage; Emergency Sheltering; Environmental Sustainability; Equity; Extreme Climate; Facilities for the Elderly; Flood Prevention; Food Safety; Green Building; Green Infrastructure; Green Space; Green Transportation; Greenway; Harmless Garbage; Health Promotion; Health Promotion Activity; Health Science and Technology; Health Surveillance; Healthcare; Health Services; Healthy Behavior; Healthy City; Healthy Food; Healthy Society; Healthy Workplace; Jogging; Livability; Low Carbon; Mental Health; Obesity Prevention; Park; Physical Exercise; Playground; Plaza; Pollution Control; Positive Aging; Public Engagement; Public Health; Public Space; Public Transportation; Quality of Life; Resilience; Safe Community; Sewage Treatment; Social Inclusion; Sponge City; Sports; Sports Facility; Stress Reduction; Sustainable Development; Transportation Safety; Urban Heat Island Effect; Walking; Walking-Friendly; Waste Management; Waste Reduction; Water Quality; Waterlogging Removal; Well-being
